# Rome III Criteria Capture Higher Irritable Bowel Syndrome SNP-Heritability and Highlight a Novel Genetic Link with Cardiovascular Traits

**DOI:** 10.1016/j.jcmgh.2024.04.002

**Published:** 2024-04-19

**Authors:** Leticia Camargo Tavares, Esteban Alexander Lopera-Maya, Ferdinando Bonfiglio, Tenghao Zheng, Trishla Sinha, Francine Zanchetta Marques, Alexandra Zhernakova, Serena Sanna, Mauro D’Amato

**Affiliations:** 1 School of Biological Sciences, Monash University, Clayton, Australia; 2 Department of Genetics, University of Groningen, University Medical Center Groningen, Groningen, the Netherlands; 3 Department of Chemical, Materials and Production Engineering, University of Naples Federico II, Naples, Italy; 4 CEINGE Biotecnologie Avanzate s.c.ar.l., Naples, Italy; 5 Heart Failure Research Group, Baker Heart and Diabetes Institute, Melbourne, Australia; 6 Institute for Genetic and Biomedical Research, National Research Council, Cagliari, Italy; 7 Gastrointestinal Genetics Lab, CIC bioGUNE – BRTA, Derio, Spain; 8 Ikerbasque, Basque Foundation for Science, Bilbao, Spain; 9 Department of Medicine and Surgery, LUM University, Casamassima, Italy

**Keywords:** Genome-Wide Association Study, IBS, Genetic Correlation, CVD

## Abstract

**Background & Aims:**

Irritable bowel syndrome (IBS) shows genetic predisposition, and large-scale genome-wide association studies (GWAS) are emerging, based on heterogeneous disease definitions. We investigated the genetic architecture of IBS defined according to gold standard Rome Criteria.

**Methods:**

We conducted GWAS meta-analyses of Rome III IBS and its subtypes in 24,735 IBS cases and 77,149 asymptomatic control subjects from 2 independent European cohorts (UK Biobank and Lifelines). Single-nucleotide polymorphism (SNP)-based heritability (h^2^_SNP_) and genetic correlations (r_g_) with other traits were calculated. IBS risk loci were functionally annotated to identify candidate genes. Sensitivity and conditional analyses were conducted to assess impact of confounders. Polygenic risk scores were computed and tested in independent datasets.

**Results:**

Rome III IBS showed significant SNP-heritability (up to 13%) and similar genetic architecture across subtypes, including those with manifestations at the opposite ends of the symptom spectrum (r_g_ = 0.48 between IBS-D and IBS-C). Genetic correlations with other traits highlighted commonalities with family history of heart disease and hypertension, coronary artery disease, and angina pectoris (r_g_ = 0.20–0.45), among others. Four independent GWAS signals (*P* < 5×10^-8^) were detected, including 2 novel loci for IBS (rs2035380) and IBS-mixed (rs2048419) that had been previously associated with hypertension and coronary artery disease. Functional annotation of GWAS risk loci revealed genes implicated in circadian rhythm (*BMAL1*), intestinal barrier (*CLDN23*), immunomodulation (*MFHAS1*), and the cyclic adenosine monophosphate pathway (*ADCY2*). Polygenic risk scores allowed the identification of individuals at increased risk of IBS (odds ratio, 1.34; *P* = 1.1×10^-3^).

**Conclusions:**

Rome III Criteria capture higher SNP-heritability than previously estimated for IBS. The identified link between IBS and cardiovascular traits may contribute to the delineation of alternative therapeutic strategies, warranting further investigation.

## Graphical abstract

**Figure:**
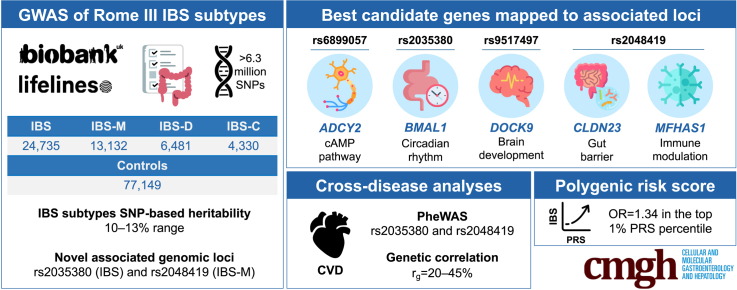

**Summary**

Genome-wide association studies on irritable bowel syndrome defined according to consensus Rome criteria shows higher single-nucleotide polymorphism–based heritability than previously reported, and novel risk loci with attractive druggable targets. Genetic profiling shows overlap with cardiovascular diseases and may contribute to the stratification of patients with irritable bowel syndrome.

Irritable bowel syndrome (IBS) is a common disorder of gut–brain interaction.^1^ It affects women more often than men and has a prevalence of 3%–11% in European populations, based on the consensus Rome criteria from the Rome Foundation.^2^ These criteria define IBS as a recurrent abdominal pain or discomfort associated with abnormal stool frequency and consistency, and classify IBS subtypes as constipation (IBS-C), diarrhea (IBS-D), or mixed (IBS-M) based on the predominant stool type.^3^ The latest Rome IV criteria are the most stringent, focusing only on abdominal pain (rather than also discomfort, as in Rome III) and an increased frequency of symptoms (1 day/week vs 3 days/month in Rome III), thus possibly capturing a more severe phenotype compared with earlier versions.^4^^,^^5^

Existing treatments for IBS are mostly directed toward the amelioration of symptoms, but are not effective in all patients.^6^ The contribution of dietary and psychological factors, gut dysbiosis, prior gastrointestinal (GI) infection, and genetic predisposition is recognized.^1^ A heritable component of IBS has been demonstrated in twin and family studies, although with varying degrees of estimates depending on the adopted IBS definition.^7^ The highest heritability was estimated in the Australian Twin study, with 57.9% (95% confidence interval, 40.6–75.9) of the variance in the reporting of IBS symptoms being attributed to additive genetic effects.^8^ Whether these genetic factors differently predispose to various IBS forms and/or subtypes is unclear, which hampers personalized approaches to treat patients with distinct clinical presentations.^6^

Interest in the genetic architecture of IBS has been growing, because it holds potential to reveal actionable pathways and biomarkers.^9^ This can be sought, for instance, by analogy with other diseases showing similar pathophysiologic mechanisms, including those accounted for by shared genetic factors. Despite demonstrated heritability, however, previous IBS candidate-gene studies lacked power and replication, except for serotonin, sucrase-isomaltase and ion channel genes.^10^, ^11^, ^12^, ^13^ Recently, well-powered genome-wide association studies (GWAS) of IBS and endophenotypes (eg, gut motility) have emerged, which made use of genotypic and health-related data from large cohorts and population-based biobanks.^9^^,^^14^, ^15^, ^16^, ^17^ However, these studies used heterogeneous IBS definitions that show poor overlap and inconsistent findings across definitions. For instance, the largest GWAS meta-analysis included results from 53,400 IBS cases, collectively identified through hospital-inpatient records, self-reported diagnoses, and Rome criteria: among those included from the UK Biobank (UKBB), less than 1% (n = 340/40,548) satisfied all IBS definitions simultaneously.^15^ The applicability and clinical relevance of genetic findings obtained using pooled IBS definitions are yet to be determined, nor have different IBS manifestations been adequately explored based on stratification of patients in different Rome subtype groups.

Here, we investigate the genetic underpinnings of IBS and its subtypes, based on available genotype and Rome III questionnaire data from 2 large European population-based biobanks (UKBB and Lifelines [LL]), totaling 24,735 cases and 77,149 asymptomatic control subjects.

## Results

### Characteristics of Rome III IBS Patients

**Figure 1:**
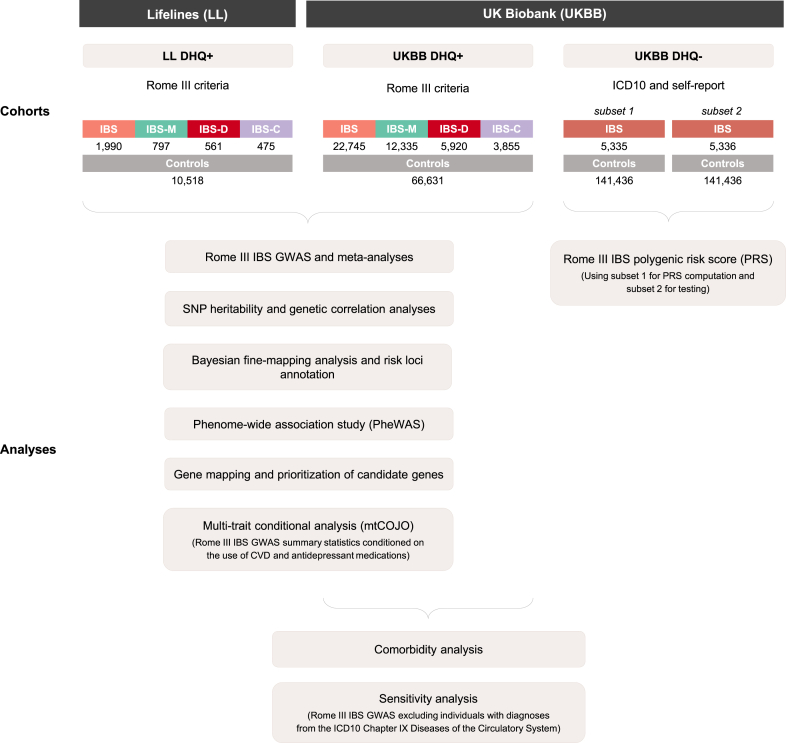
**Infographic summarizing the derivation of study cohorts (IBS cases and control subjects) and their inclusion in different analyses.** Digestive health questionnaire (DHQ) responders are described as “DHQ+” and nonresponders as “DHQ-.”

Rome III IBS patients and asymptomatic control subjects were defined based on digestive questionnaire data available for UKBB and LL participants (see Methods, Figure 1). In total, we identified 24,735 Rome III IBS patients (22,745 UKBB patients and 1990 LL patients) and 77,149 asymptomatic control subjects (66,631 from UKBB and 10,518 from LL). The demographics of study participants are summarized in Table 1. In line with previous studies, IBS-M was the most common subtype both in UKBB (54% of patients with IBS) and LL (40%). Patients with IBS were predominantly women, with the IBS-C group showing the most pronounced female predominance (84.0% of IBS-C cases were female in both UKBB and LL).^18^^,^^19^

**Table 1:** Demographics of Study Cohorts Included in Rome III IBS GWAS Meta-analyses

Cohort		N	Age, mean (SD)	Sex, female (*%*)
UKBB	IBS	22,745	54.4 (7.7)	73.0
	IBS-M	12,335	56.6 (7.6)	72.4
	IBS-D	5,920	54.2 (7.7)	67.4
	IBS-C	3,855	55.0 (7.8)	83.9
	Control subjects	66,631	56.6 (7.6)	46.9
LL	IBS	1,990	42.5 (14.0)	76.2
	IBS-M	797	40.4 (14.2)	78.9
	IBS-D	561	43.1 (13.4)	67.9
	IBS-C	475	44.0 (14.1)	84.0
	Control subjects	10,518	47.2 (13.8)	48.0

GWAS, genome-wide association study; IBS, irritable bowel syndrome; IBS-C, irritable bowel syndrome constipation-predominant subtype; IBS-D, irritable bowel syndrome diarrhea-predominant subtype; IBS-M, irritable bowel syndrome mixed subtype; LL, Lifelines; SD, standard deviation; UKBB, UK Biobank.

**Figure 2:**
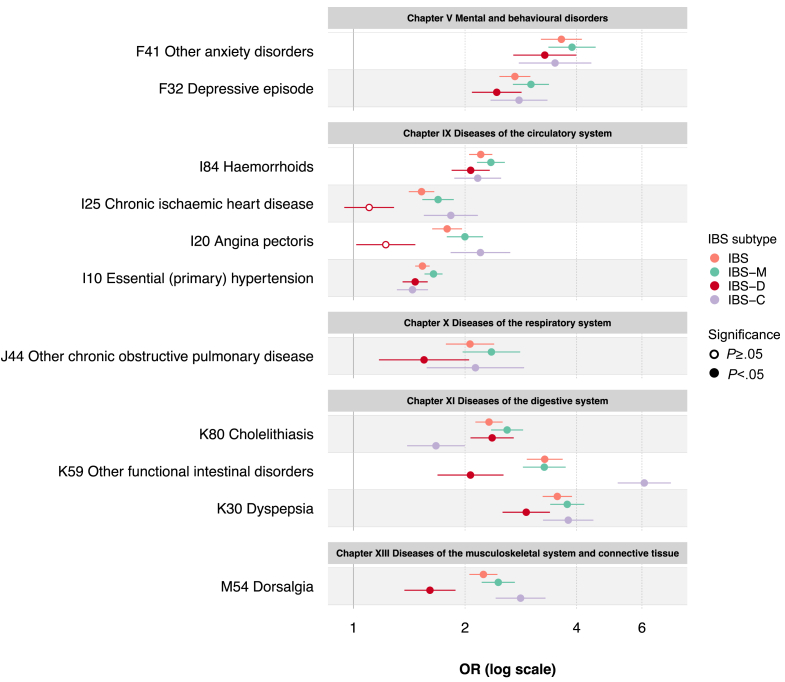
**Analysis of Rome III IBS comorbidities in UKBB.** Selected ICD10 diagnoses at the 3-digit code (trait-specific) level are reported, with corresponding OR and 95% confidence interval versus asymptomatic control subjects, derived from logistic regression adjusted for sex and age. Nonsignificant OR (p_FDR_ ≥ .05) are shown as *empty circles*. Full results are reported in Supplementary Table 1.

Health-related data available in UKBB allowed other diseases and conditions to be studied in IBS cases and control subjects, based on hospital-inpatient records in relation to diseases (International Classification of Diseases, 10th version [ICD10], Chapters I-XIV) and laboratory findings (ICD10 Chapter XVIII), across 22,344,000 data-points (250 data-fields covering 89,376 individuals) (Methods). Age- and sex-adjusted regression analysis revealed patients with IBS (all subtypes) to suffer from a significantly higher number of comorbidities compared with control subjects (*P* ≤ 5.1×10^-161^) (Table 2). At both the chapter (domain) and 3-digit (trait-specific) ICD10 levels, strongest associations were detected for GI (chapter XI *diseases of the digestive system*) and psychiatric (chapter V *mental and behavioral disorders*) disorders, whose risk was more than doubled in Rome III IBS versus control subjects (odds ratio [OR], 2.0–2.7; *P* ≤ 7.7×10^-39^) (Table 3), particularly for F41 *other anxiety disorders* (OR, 3.3–3.9; *P* ≤ 4.5×10^-26^) and K30 *dyspepsia* (OR, 2.9–3.8; *P* ≤ 1.0×10^-44^) (Figure 2, Supplementary Table 1). As a novel observation, traits from the chapter IX *diseases of the circulatory system* showed increased prevalence among patients with IBS (Figure 2, Supplementary Table 1). This included I10 *essential (primary) hypertension*, which was associated with all IBS subtypes (OR, 1.4–1.6; *P* ≤ 1.3×10^-12^), more pronouncedly IBS-M (Figure 2). Other differences, also related to diseases of the circulatory system, were observed across subtypes including I20 *angina pectoris* and I25 *chronic ischemic heart disease*, which were more common in IBS-C patients (OR, 2.2 [*P* = 1.2×10^-15^] and OR, 1.8 [*P* = 2.1×10^-11^], respectively) (Figure 2, Supplementary Table 1).

**Table 2:** Number of ICD10 Comorbidities in UKBB Subjects

Cohort	Number of ICD10 comorbidities		
	Mean (SD)	Beta (SE)	*P* (Beta)^a^
IBS	4.5 (5.1)	1.81 (0.03)	<1.0E-300
IBS-M	4.7 (5.3)	2.02 (0.04)	<1.0E-300
IBS-D	4.1 (4.6)	1.42 (0.05)	5.1E-161
IBS-C	4.6 (5.3)	1.93 (0.06)	1.9E-194
Control subjects	3.0 (3.9)	—	—

IBS, irritable bowel syndrome; IBS-C, irritable bowel syndrome constipation-predominant subtype; IBS-D, irritable bowel syndrome diarrhea-predominant subtype; IBS-M, irritable bowel syndrome mixed subtype; ICD10, International Classification of Diseases, 10th revision; SD, standard deviation; SE, standard error; UKBB, UK Biobank.

a From sex- and age-adjusted linear regression, testing the effect of IBS on the number of ICD10 comorbidities. Comorbidities included ICD10 codes for diseases (Chapters I-XIV) and laboratory findings (Chapter XVIII), excluding K58 for IBS and confounding gastrointestinal diagnoses (see Methods).

**Table 3:** Chapter-Level ICD10 Comorbidities in UKBB Subjects

ICD10 Chapter	OR (95% CI)				*P* value (FDR)^a^			
	IBS	IBS-M	IBS-D	IBS-C	IBS	IBS-M	IBS-D	IBS-C
I Certain infectious and parasitic diseases	2.1 (1.9–2.2)	2.2 (2.1–2.4)	2.0 (1.8–2.2)	1.9 (1.6–2.1)	5.0E-104	1.4E-87	6.5E-35	4.2E-19
II Neoplasms	1.2 (1.2–1.3)	1.2 (1.2–1.3)	1.3 (1.2–1.3)	1.3 (1.2–1.4)	3.5E-28	3.2E-17	3.0E-10	4.4E-10
III Diseases of the blood and blood-forming organs and certain disorders involving the immune mechanism	1.5 (1.4–1.7)	1.6 (1.5–1.8)	1.4 (1.2–1.6)	1.6 (1.4–1.9)	3.1E-30	3.8E-27	9.5E-06	6.0E-10
IV Endocrine, nutritional and metabolic diseases	1.6 (1.5–1.7)	1.8 (1.7–1.9)	1.5 (1.4–1.6)	1.5 (1.4–1.7)	2.0E-100	3.2E-98	3.3E-22	9.6E-18
V Mental and behavioral disorders	2.2 (2.1–2.4)	2.4 (2.3–2.6)	2.0 (1.8–2.2)	2.3 (2.0–2.6)	4.1E-140	2.3E-120	7.7E-39	3.3E-39
VI Diseases of the nervous system	1.7 (1.6–1.8)	1.8 (1.7–1.9)	1.5 (1.4–1.7)	1.8 (1.6–2.0)	4.4E-80	1.2E-63	3.3E-18	2.3E-27
VII Diseases of the eye and adnexa	1.2 (1.1–1.3)	1.2 (1.1–1.3)	1.2 (1.0–1.3)	1.2 (1.0–1.3)	8.3E-10	4.7E-08	1.4E-02	3.8E-02
VIII Diseases of the ear and mastoid process	1.5 (1.4–1.7)	1.6 (1.4–1.8)	1.5 (1.3–1.8)	1.6 (1.3–2.0)	8.2E-15	2.8E-11	1.8E-05	3.0E-05
IX Diseases of the circulatory system	1.6 (1.6–1.7)	1.7 (1.7–1.8)	1.5 (1.4–1.6)	1.6 (1.5–1.7)	1.3E-146	7.5E-125	1.3E-33	7.5E-33
X Diseases of the respiratory system	1.7 (1.6–1.8)	1.8 (1.7–1.9)	1.5 (1.4–1.6)	1.7 (1.6–1.9)	5.2E-106	2.8E-90	2.4E-23	1.9E-28
XI Diseases of the digestive system	2.5 (2.4–2.6)	2.7 (2.5–2.8)	2.3 (2.2–2.4)	2.6 (2.4–2.8)	<1.0E-300	<1.0E-300	1.5E-178	1.8E-157
XII Diseases of the skin and subcutaneous tissue	1.3 (1.2–1.4)	1.3 (1.2–1.4)	1.3 (1.2–1.4)	1.3 (1.1–1.4)	1.3E-19	1.8E-16	3.0E-06	1.2E-04
XIII Diseases of the musculoskeletal system and connective tissue	1.5 (1.4–1.5)	1.6 (1.5–1.6)	1.3 (1.3–1.4)	1.5 (1.4–1.7)	1.2E-102	1.0E-85	2.0E-20	3.4E-30
XIV Diseases of the genitourinary system	1.5 (1.5–1.6)	1.6 (1.6–1.7)	1.4 (1.3–1.5)	1.7 (1.5–1.8)	1.0E-123	3.3E-101	3.6E-28	6.4E-41
XVIII Symptoms, signs and abnormal clinical and laboratory findings, not elsewhere classified	2.1 (2.1–2.2)	2.3 (2.2–2.4)	2.0 (1.9–2.1)	2.2 (2.0–2.4)	<1.0E-300	<1.0E-300	1.4E-119	1.8E-108

CI, confidence interval; FDR, false discovery rate; IBS, irritable bowel syndrome; IBS-C, irritable bowel syndrome constipation-predominant subtype; IBS-D, irritable bowel syndrome diarrhea-predominant subtype; IBS-M, irritable bowel syndrome mixed subtype; ICD10, International Classification of Diseases, 10th revision; OR, odds ratio; SD, standard deviation; SE, standard error; UKBB, UK Biobank.

a From sex- and age-adjusted logistic regressions, comparing the risk of ICD10 diagnoses (at the chapter level) in IBS cases versus control subjects. Nonsignificant results (FDR >0.05) are omitted (indicated by a dash). Only ICD10 chapters for diseases (Chapters I-XIV) and laboratory findings (Chapter XVIII) are included, excluding K58 for IBS and confounding gastrointestinal diagnoses (see Methods).

### Rome III IBS GWAS Meta-Analyses: Heritability and Genetic Correlation with Other Traits

**Figure 3:**
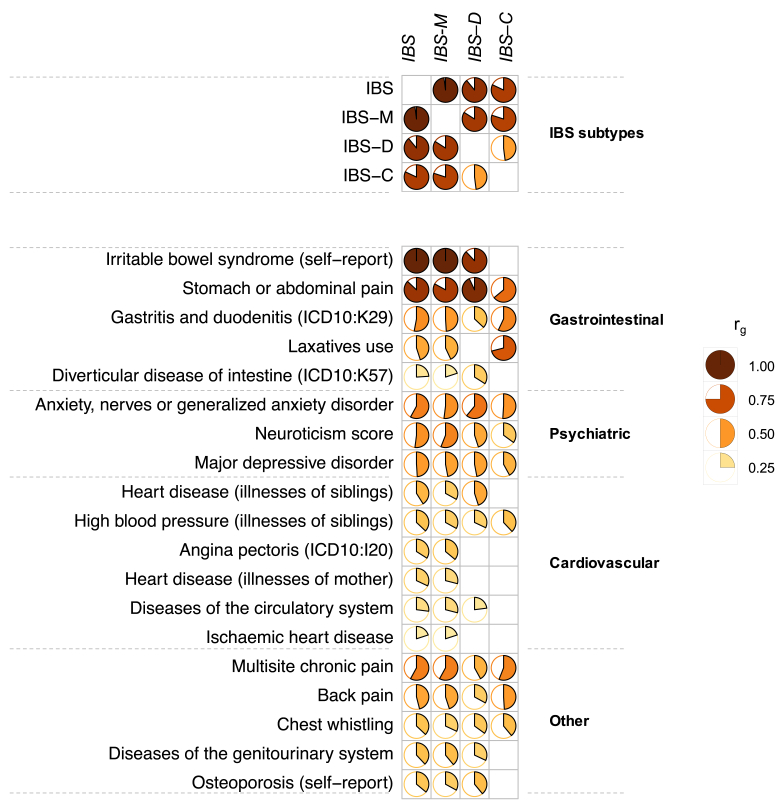
**Genetic correlations (r**_**g**_**) across Rome III IBS subtypes and other traits.** IBS, its subtypes, and other traits are reported based on respective significant (p_FDR_ < .05) genetic correlations, divided into different domains. *Empty cells* correspond to nonsignificant results or r_g_ between the same traits. Full results are reported in Supplementary Table 2.

Individual Rome III IBS GWAS analyses were carried out in UKBB and LL, adopting a common analytic pipeline for quality control (per-sample and per-marker), imputation, and association testing adjusting for age, sex, and principal components (PCs) (Methods). UKBB and LL GWAS summary statistics for 6,311,313 high-quality single-nucleotide polymorphism (SNP) markers were included in GWAS meta-analyses spanning, respectively, 24,735 IBS, 13,132 IBS-M, 6481 IBS-D, and 4330 IBS-C cases and a common set of 77,149 asymptomatic control subjects (Table 1). GWAS meta-analysis results showed no population stratification (λ=1.03–1.09, linkage disequilibrium score regression [LDSC], intercept=0.97–1.00). Based on LDSC analyses (Methods), all Rome III IBS subtypes showed detectable and significant SNP-based heritability (h^2^_SNP_), estimated at 10.3% (*P* = 7.1×10^-25^) for IBS, 13.4% (*P* = 3.2×10^-15^) for IBS-M, 12.4% (*P* = 1.7×10^-8^) for IBS-D, and 11.7% (*P* = 1.6×10^-4^) for IBS-C. Considerable genetic overlap was observed across all traits, with almost complete correlation between IBS and IBS-M (r_g_=0.98; *P* < 1.0×10^-300^) (Figure 3, Supplementary Table 2). Of note, although weaker than with other subtypes, positive genetic correlation was also observed between traits at the opposite ends of the stool consistency spectrum, such as IBS-C and IBS-D (r_g_=0.48; *P* = 5.6×10^-3^).

The genetic architecture of IBS was then compared with that of other traits and diseases via LDSC analyses implemented in the Complex Traits Genetics Virtual Lab (CTG-VL) platform (Methods), which revealed significant correlations (p_FDR_ < .05) between IBS types and 278 other traits across the GI, psychiatric, musculoskeletal, respiratory, cardiovascular, and metabolic domains (full results in Supplementary Table 2). Strongest correlations were observed for self-reported IBS and stomach or abdominal pain (Figure 3). Indeed, among different IBS types, IBS-C is the one that most often departed from others in its genetic similarities with other traits, illustrated for instance by reduced or augmented genetic correlations with stomach or abdominal pain, laxatives, and back pain (Figure 3). Among non-GI and non-pain-related traits, the conditions most closely correlating with IBS were psychiatric conditions, including generalized anxiety disorder (r_g_=0.51–0.61; *P* = ≤ 2.2×10^-2^), major depressive disorder (r_g_=0.42–0.49; p≤1.9×10^-5^), and neuroticism score (r_g_=0.35–0.56; *P* ≤ 4.1×10^-4^), all 3 showing stronger mean correlations with IBS-M and IBS-D than IBS-C. As an interesting and new observation, significant genetic correlations with IBS (particularly the IBS-M subtype) were noted for cardiovascular diseases (CVD), including family history of heart disease (measured as illness of mother and of siblings; r_g_=0.29–0.45; *P* ≤ 1.3×10^-3^) and high blood pressure (BP, measured as illness of siblings; r_g_=0.32–0.38; *P* ≤ 2.2×10^-2^), among others (Figure 3, Supplementary Table 2).

### Annotation of Genomic Risk Loci and Fine Mapping

**Figure 4:**
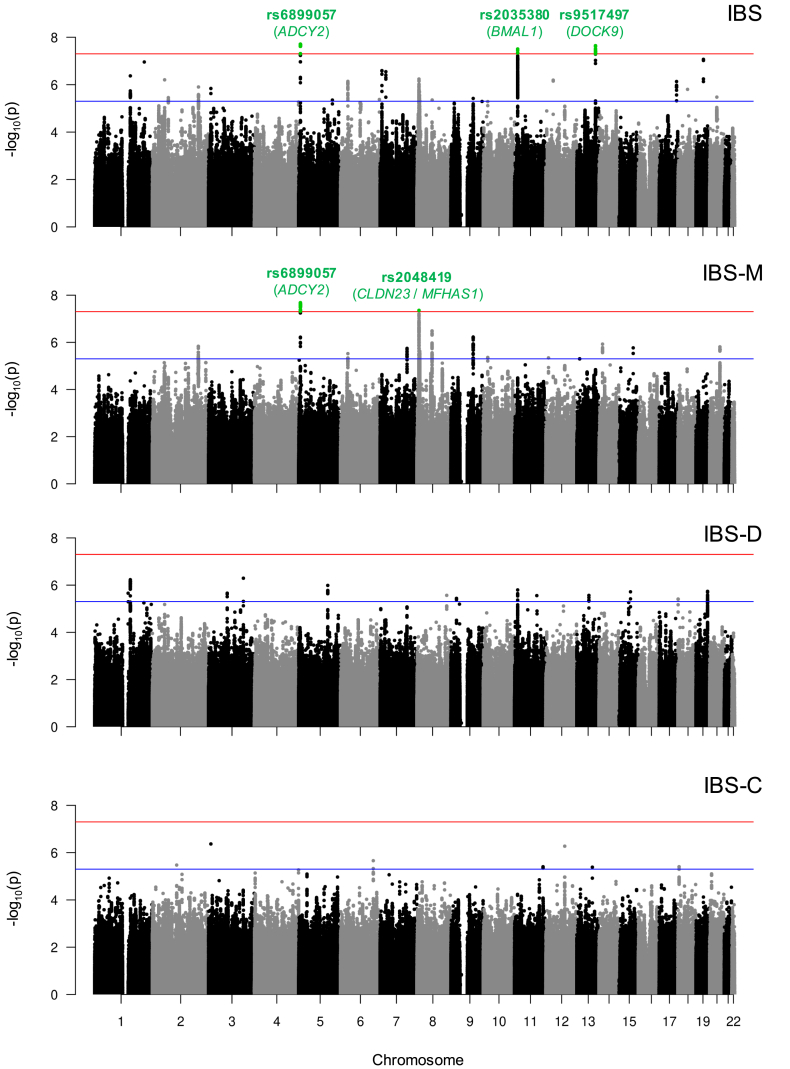
**Manhattan plots of subtype-specific IBS ΩGWAS meta-analyses.** Each *circle* denotes a marker with regard to its physical location (based on the Genome Reference Consortium Human Build 37) and associated -log_10_*P* value. SNPs reaching the genome-wide significance threshold (*P* = 5×10^-8^, indicated with a *horizontal red line*) are colored in *green*. Suggestive significance threshold (*P* = 5×10^-6^) is indicated with a *horizontal blue line*. The lead SNP rs ID and its respective best candidate protein-coding gene symbol are annotated in *green*.

**Figure 5:**
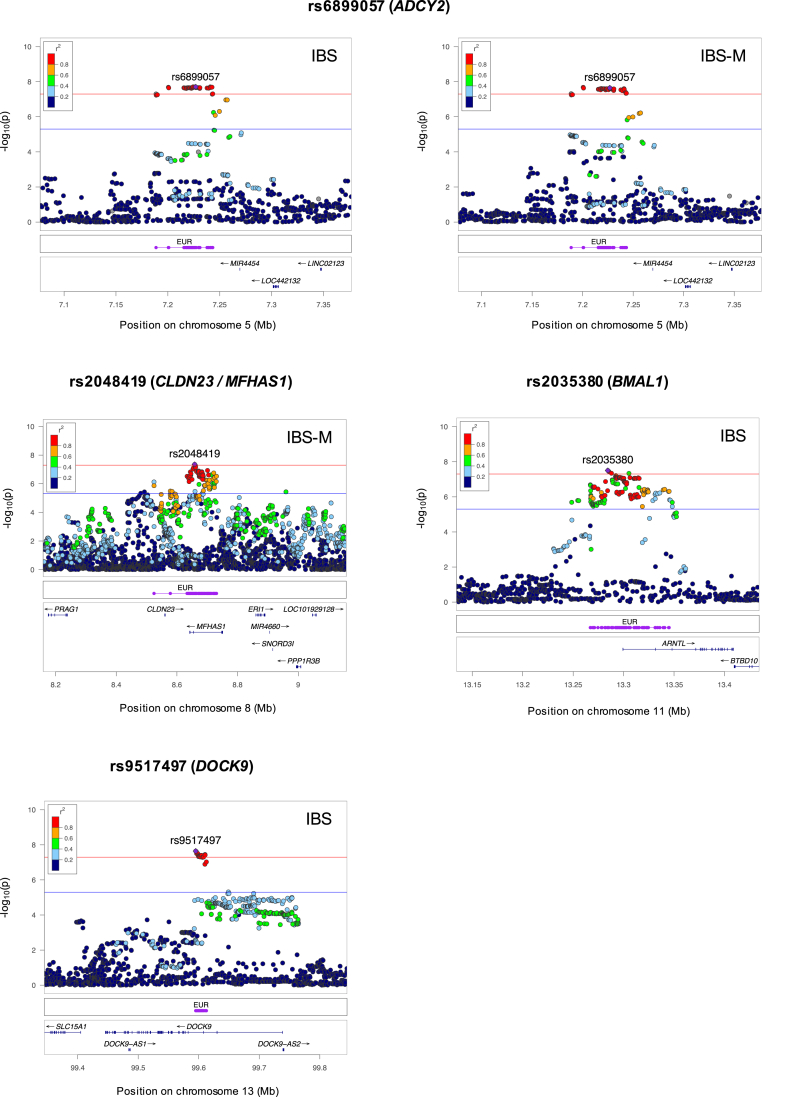
**Regional plots of associated IBS and IBS-M risk loci.** SNPs (*dots*) are reported with their physical location (based on the Genome Reference Consortium Human Build 37), associated -log_10_*P* value, and degree of linkage disequilibrium (*r*^2^) with the lead SNP (*purple diamond* labelled with the rs ID). The *red line* indicates the genome-wide threshold at *P* = 5×10^-8^ and the *blue line* indicates the suggestive threshold at *P* = 5×10^-6^. *Purple lines* labelled as “EUR” indicate the genomic location of fine-mapped credible variants.

Sixty-four genome-wide significant SNP associations (*P* ≤ 5×10^-8^) were detected in Rome III IBS GWAS meta-analyses, corresponding to 4 independent loci: 2 in IBS (tagged by rs2035380 and rs9517497 on chromosome 11 and 13), 1 in IBS-M (tagged by rs2048419 on chromosome 8), and 1 common to IBS and IBS-M (tagged by the lead SNP rs6899057 on chromosome 5), as shown in Table 4 and Figure 4. No genome-wide significant associations were detected for IBS-D or IBS-C (Figure 4). Association signals from the 4 Rome IBS risk loci (regional plots in Figure 5) showed no heterogeneity (Cochran P-Het>0.05) and consistent genetic risk effects (OR direction) across GWAS performed individually in UKBB and LL (Table 4). Notably, rs2035380 (candidate gene *BMAL1*) and rs2048419 (*CLDN23*/*MFHAS1*) represent novel associations identified in this study, respectively, for IBS and the IBS-M subtype. In addition, although genome-wide associations for rs6899057 (*ADCY2*) and rs9517497 (*DOCK9*) had been previously reported for IBS,^10^ the former is also linked here to IBS-M for the first time.

**Table 4:** Rome III IBS Risk Loci Identified Via GWAS Meta-analyses

CHR	Lead SNP	Start–end (BP)	EA	OA	EAF	GWAS					Credible Variants, n	Gene mapping	Novel risk locus
						IBS subtype	OR (95% CI)	*P* value	OR in individual cohorts (UKBB/LL)	P-Het			
5	rs6899057	7188657–7257239	T	C	0.36	IBS	1.07 (1.04–1.09)	2.0E-08	1.07/1.03	0.40	41	***ADCY2***^*e*^	x
						IBS-M	1.09 (1.05–1.12)	2.3E-08	1.08/1.09	0.97			
8	rs2048419	8524474–8730488	A	G	0.53	IBS-M	1.08 (1.05–1.11)	4.4E-08	1.08/1.03	0.34	77	***CLDN23***^*e*^ *ERI1*^*e*^ ***MFHAS1***^*p,e*^ *PPP1R3B*^*e*^ *TNKS*^*e*^	✓
11	rs2035380	13267867–13346294	T	C	0.30	IBS	1.07 (1.04–1.09)	3.1E-08	1.07/1.04	0.46	99	***BMAL1***^*p,e*^	✓
13	rs9517497	99594682–99612588	T	C	0.63	IBS	1.07 (1.04–1.09)	2.3E-08	1.06/1.09	0.48	20	***DOCK9***^*p,e*^	x

BP, base-pair position (genome build hg19); CHR, chromosome; CI, confidence interval; EA, effect allele; EAF, effect allele frequency; GWAS, genome-wide association study; IBS, irritable bowel syndrome; IBS-M, irritable bowel syndrome mixed subtype; LL, Lifelines; OA, other allele; OR, odds ratio; SNP, single-nucleotide polymorphism; UKBB, UK Biobank.

OR in individual cohorts: magnitude of mean effects described to the risk allele in UKBB and LL individual GWAS; P-Het: Cochran's Q statistics *P* value for heterogeneity across individual GWAS; Number of credible variants: number of markers in each fine-mapped credible set per risk locus; Gene mapping: genes mapped to the locus, based on FUMA positional (^p^) or cis-eQTL (^e^) analyses. The best candidate genes at each locus are highlighted in bold.

**Figure 6:**
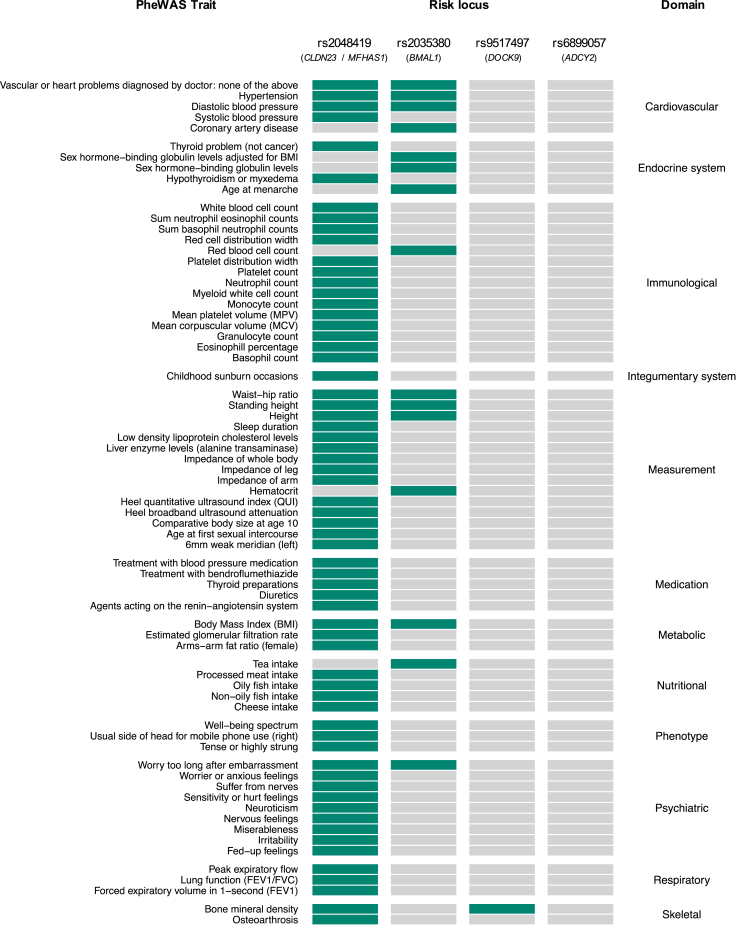
**Phenome-wide association (PheWAS) results for IBS and IBS-M risk loci.** Known GWAS associations (*P* ≤ 5×10^-8^) for lead SNPs and/or their LD proxies (*R*^2^ >0.8) were extracted from OpenTargets, PhenoScanner v2, and GWAS ATLAS (see Supplementary Methods). Domains were harmonized according to GWAS ATLAS classifications and are sorted alphabetically.

To gain initial biologic insight, we adopted a phenome-wide association study approach to screen Rome III IBS risk loci for known associations (*P* ≤ 5×10^-8^) reported in previous GWAS studies (Methods). Similar to the results obtained from genetic correlations, these analyses revealed the 2 novel risk loci, tagged by rs2048419 (*CLDN23*/*MFHAS1*) and rs2035380 (*BMAL1*), had both been previously associated with multiple traits including cardiovascular (hypertension, coronary artery disease), psychiatric (anxiety-like), and metabolic (body mass index) traits (Figure 6).

**Figure 7:**
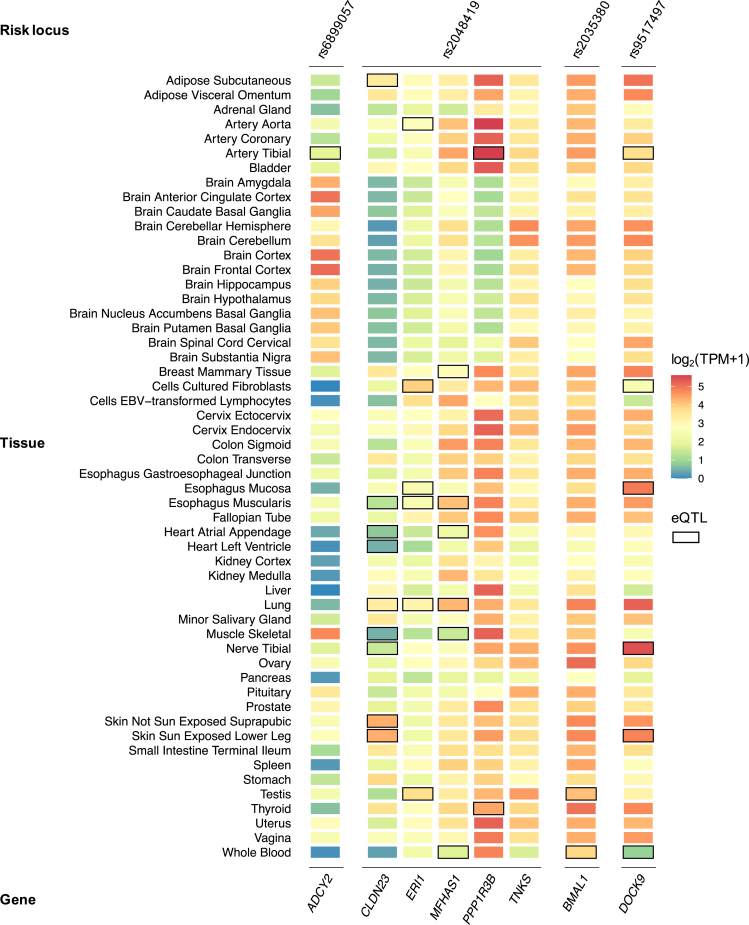
**Heat map of gene expression from Rome III IBS risk loci, based on GTEx data.** IBS candidate genes from IBS risk loci (locus membership annotated at the *top*) are reported with their level of expression (mRNA) in 54 human tissues using data from GTEx v8 (https://gtexportal.org). The log_2_(TPM+1), where TPM (transcripts per million) is the averaged expression per tissue and per gene, is indicated on a color-coded scale. eQTLs (p_FDR_ < .05) linked to lead SNP or variants in high LD (*r*^2^ >0.8) are *boxed*.

A Bayesian approach to fine-mapping was used to try and identify the most likely causative variants from each locus (Methods). This returned 4 independent credible sets of SNPs (range, 20–99 markers each) with high posterior probabilities to include causative variants (posterior probability, 82.0%–91.4%). However, no individual variant was estimated to be causative with high confidence (all variants were lower than 8.0% probability), and the identity of the individual causal variant from each locus remained elusive. Although there was no coding variant among SNPs from the credible set of each locus, several mapped near regulatory regions and transcription factors binding sites (Table 5). Furthermore, many were associated with differential expression (expression quantitative trait loci [eQTL]) of multiple genes in various tissues from the Genotype-tissue Expression (GTEx) Portal (Figure 7; see Methods), including eQTLs in tissues of the GI tract (for rs2048419 and rs9517497 loci) and heart (for rs2048419).

**Table 5:** List of Fine-Mapped Credible Variants That Are Near Regulatory Sites Based on Ensembl Variation Annotation

Lead SNP	Credible SNP	Biotype	CHR	BP	EA	OA	EAF	GWAS		
								IBS subtype	OR (95% CI)	*P*
**rs6899057** *ADCY2*	rs4286635	Enhancer	5	7242559	A	C	0.60	IBS	0.94 (0.92–0.96)	2.0E-08
	rs4286635	Enhancer	5	7242559	A	C	0.60	IBS-M	0.92 (0.90–0.94)	4.1E-08
	rs7718889	CTCF binding site	5	7200526	A	T	0.63	IBS	0.94 (0.92–0.96)	2.1E-08
	rs7718889	CTCF binding site	5	7200526	A	T	0.63	IBS-M	0.92 (0.90–0.94)	2.1E-08
**rs2048419** *CLDN23* / *MFHAS1*	rs1039915	Enhancer	8	8679614	T	C	0.53	IBS-M	1.07 (1.05–1.09)	2.3E-07
	rs1039916	Enhancer	8	8685854	A	G	0.52	IBS-M	1.07 (1.05–1.09)	2.7E-07
	rs1039917	Enhancer	8	8718850	A	G	0.37	IBS-M	0.93 (0.91–0.95)	9.6E-07
	rs11775523	Enhancer	8	8679176	A	G	0.53	IBS-M	1.07 (1.05–1.09)	2.7E-07
	rs11992186	CTCF binding site	8	8524474	C	G	0.49	IBS-M	1.07 (1.05–1.09)	1.4E-06
	rs13270070	Enhancer	8	8691622	A	T	0.44	IBS-M	0.93 (0.91–0.95)	1.4E-06
	rs13282015	Enhancer	8	8659675	T	G	0.76	IBS-M	0.92 (0.88–0.96)	3.4E-06
	rs2409092	Enhancer	8	8682192	A	T	0.53	IBS-M	1.07 (1.05–1.09)	1.5E-07
	rs332039	Enhancer	8	8723651	C	G	0.57	IBS-M	1.07 (1.05–1.09)	1.8E-06
	rs35039922	Enhancer	8	8675325	A	T	0.53	IBS-M	1.07 (1.05–1.09)	2.4E-07
	rs35900578	Enhancer	8	8719513	A	G	0.40	IBS-M	0.93 (0.91–0.95)	4.7E-07
	rs3789843	Enhancer	8	8724257	T	C	0.41	IBS-M	0.93 (0.91–0.95)	3.7E-07
	rs3789845	Enhancer	8	8723918	T	G	0.34	IBS-M	0.93 (0.91–0.95)	1.0E-06
	rs3789849	Enhancer	8	8687054	C	G	0.43	IBS-M	0.93 (0.91–0.95)	3.0E-07
	rs3827806	Enhancer	8	8724276	T	C	0.41	IBS-M	0.93 (0.91–0.95)	3.8E-07
	rs4840362	Enhancer	8	8670082	C	G	0.46	IBS-M	0.93 (0.91–0.95)	1.4E-07
	rs4841040	Promoter	8	8654527	T	C	0.53	IBS-M	1.08 (1.06–1.10)	7.8E-08
	rs4841042	Enhancer	8	8664622	A	G	0.46	IBS-M	0.93 (0.91–0.95)	1.4E-07
	rs4841051	Enhancer	8	8685646	T	C	0.52	IBS-M	1.07 (1.05–1.09)	2.2E-07
	rs560544	CTCF binding site	8	8637429	A	G	0.52	IBS-M	1.07 (1.05–1.09)	3.7E-07
	rs56367294	Enhancer	8	8722527	A	G	0.32	IBS-M	0.93 (0.89–0.97)	1.4E-06
	rs60315134	Enhancer	8	8670599	A	G	0.54	IBS-M	1.07 (1.05–1.09)	1.9E-07
	rs7820146	Enhancer	8	8699757	A	T	0.44	IBS-M	0.93 (0.91–0.95)	6.1E-07
	rs7823757	Enhancer	8	8670177	A	T	0.46	IBS-M	0.93 (0.91–0.95)	1.6E-07
	rs7833171	Enhancer	8	8699761	T	C	0.44	IBS-M	0.93 (0.91–0.95)	5.8E-07
	rs882462	Enhancer	8	8678530	A	G	0.47	IBS-M	0.93 (0.91–0.95)	3.5E-07
**rs2035380** *BMAL1*	rs10766064	Open chromatin region	11	13277932	A	G	0.74	IBS	0.94 (0.92–0.96)	5.7E-07
	rs10766065	Open chromatin region	11	13277961	T	C	0.72	IBS	0.94 (0.92–0.96)	9.4E-07
	rs10766066	Open chromatin region	11	13278027	A	G	0.72	IBS	0.94 (0.92–0.96)	9.4E-07
	rs10832018	Enhancer	11	13312424	A	G	0.71	IBS	0.94 (0.92–0.96)	8.9E-08
	rs10832021	Enhancer	11	13324530	A	G	0.71	IBS	0.94 (0.92–0.96)	5.2E-07
	rs11022733	Enhancer	11	13281557	A	C	0.71	IBS	0.94 (0.92–0.96)	8.3E-07
	rs11022734	Enhancer	11	13281580	A	G	0.29	IBS	1.06 (1.04–1.08)	8.3E-07
	rs11022735	Enhancer	11	13292727	A	C	0.30	IBS	1.06 (1.04–1.08)	7.8E-08
	rs11022743	Promoter	11	13297800	A	G	0.27	IBS	1.06 (1.04–1.08)	5.7E-07
	rs11022753	CTCF binding site	11	13310854	T	C	0.73	IBS	0.94 (0.92–0.96)	7.8E-07
	rs11022754	Enhancer	11	13313243	A	G	0.27	IBS	1.06 (1.04–1.08)	7.3E-07
	rs11022756	Enhancer	11	13315439	A	C	0.29	IBS	1.06 (1.04–1.08)	8.9E-08
	rs11022757	Enhancer	11	13322580	A	G	0.29	IBS	1.06 (1.04–1.08)	5.5E-07
	rs11605776	Enhancer	11	13318524	A	C	0.71	IBS	0.94 (0.92–0.96)	4.1E-07
	rs12290622	Enhancer	11	13314307	A	G	0.71	IBS	0.94 (0.92–0.96)	8.5E-08
	rs12361893	Promoter	11	13301114	C	G	0.30	IBS	1.06 (1.04–1.08)	1.5E-07
	rs1351525	Promoter	11	13301548	A	T	0.29	IBS	1.06 (1.04–1.08)	1.5E-07
	rs1384030	Enhancer	11	13292909	T	C	0.30	IBS	1.06 (1.04–1.08)	8.3E-08
	rs1481891	CTCF binding site	11	13310718	T	C	0.29	IBS	1.06 (1.04–1.08)	8.7E-08
	rs1481892	Promoter	11	13301921	C	G	0.71	IBS	0.94 (0.92–0.96)	1.5E-07
	rs2035380	Enhancer	11	13284345	T	C	0.30	IBS	1.07 (1.04–1.09)	3.1E-08
	rs2219998	Enhancer	11	13292864	A	G	0.57	IBS	0.94 (0.92–0.96)	1.8E-07
	rs2279284	Promoter	11	13298750	T	C	0.27	IBS	1.06 (1.04–1.08)	5.3E-07
	rs2279285	Promoter	11	13298687	T	G	0.70	IBS	0.94 (0.92–0.96)	9.4E-08
	rs2279286	Promoter	11	13298519	A	G	0.70	IBS	0.94 (0.92–0.96)	8.4E-08
	rs2279287	Promoter	11	13298485	T	C	0.30	IBS	1.06 (1.04–1.08)	8.4E-08
	rs2403661	Enhancer	11	13285281	T	G	0.30	IBS	1.07 (1.05–1.09)	3.6E-08
	rs34148132	Enhancer	11	13314475	T	C	0.29	IBS	1.06 (1.04–1.08)	8.2E-08
	rs34796300	Enhancer	11	13315205	T	C	0.43	IBS	1.06 (1.04–1.08)	2.9E-07
	rs4603287	Promoter	11	13301335	T	G	0.29	IBS	1.06 (1.04–1.08)	1.5E-07
	rs4757138	Promoter	11	13297925	A	G	0.30	IBS	1.06 (1.04–1.08)	8.8E-08
	rs4757139	Promoter	11	13300456	T	C	0.70	IBS	0.94 (0.92–0.96)	1.3E-07
	rs4757140	Promoter	11	13300540	T	G	0.70	IBS	0.94 (0.92–0.96)	1.4E-07
	rs55769038	Enhancer	11	13331808	A	G	0.59	IBS	0.94 (0.92–0.96)	6.1E-07
	rs61882122	Promoter	11	13299895	A	G	0.30	IBS	1.06 (1.04–1.08)	9.7E-08
	rs6486116	CTCF binding site	11	13319838	A	C	0.29	IBS	1.06 (1.04–1.08)	6.3E-07
	rs6486118	Enhancer	11	13323786	A	G	0.71	IBS	0.94 (0.92–0.96)	4.0E-07
	rs6486119	Enhancer	11	13323789	T	C	0.29	IBS	1.06 (1.04–1.08)	4.3E-07
	rs6486120	Enhancer	11	13324142	T	G	0.29	IBS	1.06 (1.04–1.08)	5.1E-07
	rs7114573	Enhancer	11	13323498	A	C	0.29	IBS	1.06 (1.04–1.08)	5.2E-07
	rs7125487	Enhancer	11	13282058	C	G	0.28	IBS	1.07 (1.05–1.09)	1.3E-07
	rs72867447	Promoter	11	13301875	C	G	0.43	IBS	1.06 (1.04–1.08)	3.3E-07
	rs7928655	Promoter	11	13300252	C	G	0.30	IBS	1.06 (1.04–1.08)	8.7E-08
	rs7937432	CTCF binding site	11	13320525	A	G	0.71	IBS	0.94 (0.92–0.96)	4.1E-07
	rs7938307	CTCF binding site	11	13320526	A	C	0.71	IBS	0.94 (0.92–0.96)	3.7E-07
	rs7949336	CTCF binding site	11	13319894	A	G	0.74	IBS	0.94 (0.92–0.96)	7.6E-07
	rs7951393	Enhancer	11	13318431	T	C	0.71	IBS	0.94 (0.92–0.96)	4.7E-07
	rs998089	Enhancer	11	13284111	T	C	0.27	IBS	1.06 (1.04–1.08)	4.5E-07
**rs9517497** *DOCK9*	rs6491472	Enhancer	13	99598367	A	G	0.37	IBS	0.94 (0.92–0.96)	3.1E-08
	rs7337807	Enhancer	13	99610581	A	C	0.63	IBS	1.06 (1.04–1.08)	3.8E-08
	rs7338982	Enhancer	13	99610373	T	C	0.63	IBS	1.06 (1.04–1.08)	3.6E-08
	rs7339076	Enhancer	13	99599128	C	G	0.63	IBS	1.06 (1.04–1.08)	4.1E-08
	rs7987680	Enhancer	13	99601519	T	G	0.63	IBS	1.06 (1.04–1.08)	4.7E-08
	rs9513513	Open chromatin region	13	99595622	T	C	0.63	IBS	1.06 (1.04–1.08)	2.4E-08
	rs9517497	Open chromatin region	13	99594953	T	C	0.63	IBS	1.07 (1.04–1.09)	2.3E-08
	rs9517502	Enhancer	13	99599727	A	C	0.37	IBS	0.94 (0.92–0.96)	4.5E-08
	rs9517503	Enhancer	13	99600658	A	G	0.63	IBS	1.06 (1.04–1.08)	4.6E-08
	rs9517508	Enhancer	13	99606192	T	G	0.37	IBS	0.94 (0.92–0.96)	5.2E-08
	rs9557097	Enhancer	13	99612588	T	C	0.62	IBS	1.06 (1.04–1.08)	9.4E-08

NOTE. Functional consequence of variants was performed using the Ensembl Variant Effect Predictor tool (https://grch37.ensembl.org/info/docs/tools/vep). OR: mean effects described to the risk allele in the meta-analysis.

BP, base-pair position (genome build hg19); CHR, chromosome; CI, confidence interval; EA, effect allele; EAF, effect allele frequency; GWAS, genome-wide association study; IBS, irritable bowel syndrome; IBS-M, irritable bowel syndrome mixed subtype; LL, Lifelines; OA, other allele; OR, odds ratio; SNP, single-nucleotide polymorphism; UKBB, UK Biobank.

### Gene Mapping and Prioritization of Candidate Genes

**Figure 8:**
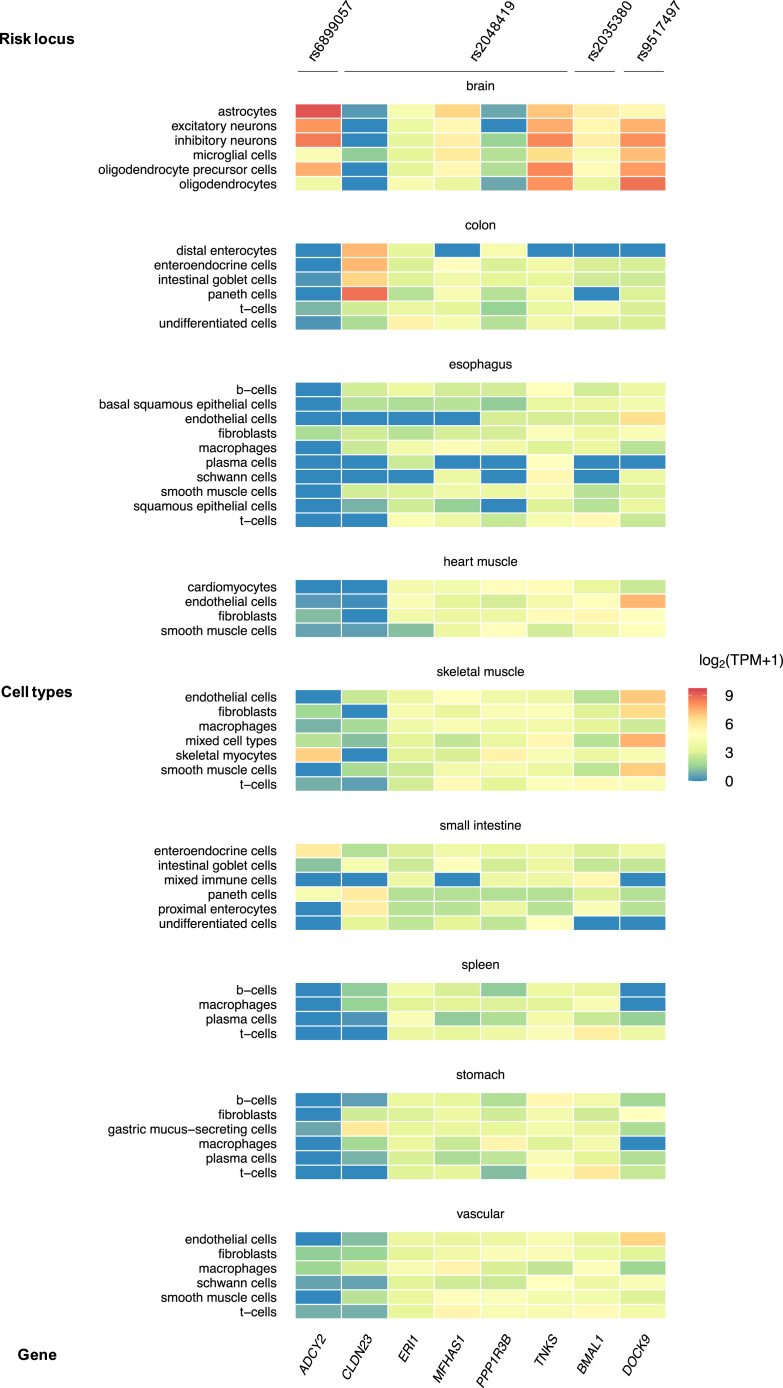
**Heat map of gene expression from Rome III IBS and IBS-M risk loci, based on single-cell transcriptomic data.** IBS candidate genes from IBS risk loci (locus membership annotated at the *top*) are reported with their level of expression (mRNA) in cell types of human tissues using single-cell transcriptomic data from the Human Protein Atlas (www.proteinatlas.org). The log_2_(TPM+1), where TPM (transcripts per million) is the averaged expression per tissue and per gene, is indicated on a color-coded scale.

Positional and cis-eQTL functional mapping (FUMA) and annotation of GWAS (Methods) resulted in a total of 8 protein-coding genes as plausible causative candidates at the 4 IBS and IBS-M risk loci (Table 4). Their expression in different human tissues and cell types are shown in Figures 7 and 8. Previous GWAS associations of these genes with various traits are listed in Supplementary Table 3.

Single genes were mapped to 3 risk loci, hence these represent bona fide candidates to be causative of the observed associations at respective loci: *BMAL1* (basic helix-loop-helix ARNT Like 1) at the new rs2035380 locus for IBS, ADCY2 (adenylate cyclase 2) at the rs6899057 locus in common for IBS and IBS-M, and *DOCK9* (dedicator of cytokinesis 9) at the rs9517497 locus for IBS (Table 1). *BMAL1*, mapped positionally and via eQTL, codes for a key clock protein that controls circadian rhythms and is highly expressed in the brain and the GI tract (colon, esophagus, small intestine, and stomach) (Figure 7), and in multiple cell types (Figure 8). This gene has been implicated via GWAS in several other traits, including CVDs and psychiatric disorders (Supplementary Table 3). DrugBank search revealed *BMAL1* regulators are targeted by antibacterial drugs with indications for IBS-D (rifaximin, DrugBank ID: DB01220) and traveler’s diarrhea (norfloxacin and ofloxacin, DrugBank ID: DB01059 and DB01165). For *ADCY2*, mapped to the rs6899057 locus via eQTLs, considerable expression is observed in brain tissues and cells (Figure 7, Figure 8) and in myocytes (Figure 8). Previous GWAS studies linked this gene to IBS and other traits, including GI diseases and CVDs, possibly via cyclic adenosine monophosphate signaling pathway (Supplementary Table 3). The encoded enzyme (AC2) is targeted by the drug colforsin indicated for smooth muscle relaxation and vasodilation (DrugBank ID: DB02587). Finally, at the rs9517497 locus, *DOCK9* is involved in GTPase activity regulation and brain development, showing high expression in brain and GI tissues (colon, esophagus, small intestine, and stomach) (Figure 7), and muscle and endothelial cell types (Figure 8). It is associated with eQTLs in the esophagus mucosa (Figure 7) and has been previously linked to IBS and many other traits via GWAS (Supplementary Table 3).

Five candidate genes were mapped to the rs2048419 locus for IBS-M, the second novel locus identified in this study (Table 4). We aimed to prioritize the best candidates at this locus based on functional evidence at multiple levels, including eQTL data for GI and brain tissues, gene expression, previous GWAS findings, mechanistic links to IBS, relevant phenotypes from knockout mice, and DrugBank data (Methods). Multiple functional annotations used for gene prioritization in the rs2048419 locus are reported in Table 6. By these means, *CLDN23* (claudin 23), which encodes a tight-junction protein that plays a role in gut barrier function, and *MFHAS1* (multifunctional ROCO family signaling regulator 1), which modulates immune responses, were deemed the most likely causative genes. Both show expression in the GI tract (Figure 7; including expression in specific colonic cell types, such as enteroendocrine cells, Figure 8), are associated with eQTLs in GI tissues via fine-mapped credible variants (Table 6), and are linked via GWAS data to other traits, such as neuroticism and hypertension (Supplementary Table 3).

**Table 6:** Functional Evidence Used for Prioritization of rs2048419 Candidate Genes

Evidence level	Candidate genes (rs2048419 locus)				
	*CLDN23* (claudin 23)	*MFHAS1* (multifunctional ROCO family signaling regulator 1)	*ERI1* (exoribonuclease 1)	*PPP1R3B* (protein phosphatase 1 regulatory subunit 3B)	*TNKS* (tankyrase)
(1) GI/brain expression	Colon, small intestine, stomach	Colon, esophagus, small intestine, stomach, brain		Colon, esophagus, small intestine, stomach	Colon, esophagus, small intestine, brain
(2) GI/brain eQTL	Esophagus	Esophagus	Esophagus		
(3) Gene-trait association	Yes (others)	Yes (others)	Yes (others)	Yes (others)	Yes (others)
(4) Mechanistic link	Tight-junction intestinal barrier function (PMID: 37798277)	Immune response modulation (PMID:28609714, 26599367, 27783989)		Lipid and glucose metabolism (PMID: 28473467)	Intestinal epithelium homeostasis (PMID: 30260955, 27190037) Immune response modulation (PMID: 35362478)
(5) Phenotype from KO model or human studies	Intestinal barrier function alterations (PMID: 37798277) Intestinal-type gastric cancer (PMID: 12736707) Immune-induced atopic dermatitis following tight-junction barrier disruption (PMID: 21163515)	Mitigation of colorectal tumors associated with inhibition of M2 macrophage polarization (PMID: 27783989)	Human intellectual disability (PMID: 36208065)	Reduced hepatic glycogen, glucose-intolerant and insulin-resistant (PMID: 28473467)	Small intestinal crypts and decreased intestinal stem cells (PMID: 30260955) Systemic inflammation (PMID: 35362478)

NOTE. Functional evidence used for gene prioritization included: (1) GI/brain expression: high expression (defined as log2TPM≥3) in GI and/or brain tissues based on GTEx data; (2) GI/brain eQTL: colocalization between a credible variant and an eQTL in a GI and/or brain tissue; (3) Gene-trait association: association with IBS or other traits in previous GWAS studies via credible variants in the gene region; (4) Mechanistic link: plausible mechanistic link between the gene and IBS; (5) Phenotype from KO model or human studies: relevant phenotype from experimental knockout/knockdown models or human studies. Obs: No genes in this locus were directly targeted by current drugs with IBS-relevant indications based on DrugBank search.

eQTL, expression quantitative trait loci; GI, gastrointestinal; GTEx, genotype-tissue expression; GWAS, genome-wide association study; IBS, inflammatory bowel syndrome.

### Genetic Link to Heart-Related Traits: Sensitivity and Conditional Analyses

Given the observed associations between IBS and heart-related traits, we sought to gain further insight from 2 additional, alternative analyses of their relationship. First, using UKBB data, we repeated our IBS Rome III GWAS analyses excluding all participants with diseases of the circulatory system (ICD10 chapter IX; diagnoses I00-I99), leaving 5993 IBS cases and 15,041 control subjects (Methods). This analysis revealed the genetic architecture of Rome III IBS to be virtually identical to that estimated devoid of heart-related conditions (r_g_>0.98 and *P* ≤ .4×10^-144^, across all Rome III IBS subtypes based on LDSC analysis of GWAS data). At the same time, the genetic risk effect estimates (ORs) associated with the 4 GWAS signals remained largely unchanged (despite the reduction in sample size and, expectedly, the strength of the associations) (Table 7). Next, we performed a proper conditional analysis to assess again, based on a different approach, whether the genetic underpinnings of Rome III IBS are independent of heart-disease predisposing factors. For this purpose, using mtCOJO^20^ (Methods), we conditioned our Rome III IBS GWAS meta-analysis results on the use of CVD medications (as proxies for their indications), using GWAS summary-level data from Wu et al^21^ (Methods). As shown in Table 8, Rome III IBS GWAS signals were not attenuated on conditioning, and the genetic architecture of IBS subtypes remained substantially unaltered, as assessed via LDSC analyses of conditioned and unconditioned GWAS data (r_g_>0.92 and *P* ≤ 7.4×10^-263^, across all Rome III IBS subtypes). Similarly, conditioning on antidepressants as proxies for anxiety and mood disorders (conditions known to share underlying genetics with IBS) did not result in substantial changes of the genetic risk effects (Table 8; and r_g_>0.94 and *P* ≤ 1.0×10^-300^ across all Rome III IBS subtypes). Overall, these results suggest that the genetic predisposition to Rome III IBS is not dependent on co-occurring heart-related diseases (and/or anxiety and mood disorders). Instead, the detected GWAS risk signals are intrinsic to the genetic architecture of IBS.

**Table 7:** Sensitivity GWAS Analysis After Removing Individuals with CVD Diagnoses in UKBB

Lead SNP	IBS subtype	Discovery^a^ IBS: 24,735 / IBS-M: 13,132 Control subjects: 77,149		UKBB ALL IBS: 22,745 / IBS-M: 12,335 Control subjects: 66,631		UKBB CVD- IBS: 16,752 / IBS-M: 8,955 Control subjects: 51,590	
		OR (95% CI)	*P* value	OR (95% CI)	*P* value	OR (95% CI)	*P* value
rs6899057 *ADCY2*	IBS	1.07 (1.04–1.09)	2.0E-08	1.08 (1.06–1.11)	6.6E-08	1.08 (1.05–1.11)	9.1E-06
	IBS-M	1.09 (1.05–1.12)	2.3E-08	1.07 (1.05–1.09)	2.0E-08	1.06 (1.04–1.09)	5.9E-06
rs2048419 *CLDN23 / MFHAS1*	IBS-M	1.08 (1.05–1.11)	4.4E-08	1.08 (1.06–1.11)	3.2E-08	1.09 (1.06–1.12)	3.2E-07
rs2035380 *BMAL1*	IBS	1.07 (1.04–1.09)	3.1E-08	1.07 (1.05–1.10)	4.0E-08	1.07 (1.04–1.10)	1.1E-06
rs9517497 *DOCK9*	IBS	1.07 (1.04–1.09)	2.3E-08	1.06 (1.04–1.09)	3.1E-07	1.06 (1.04–1.09)	8.1E-06

CI, confidence interval; CVD, cardiovascular diseases; GWAS, genome-wide association study; IBS, irritable bowel syndrome; IBS-C, irritable bowel syndrome constipation-predominant subtype; IBS-D, irritable bowel syndrome diarrhea-predominant subtype; IBS-M, irritable bowel syndrome mixed subtype; LL, Lifelines; OR, odds ratio; SD, standard deviation; SNP, single-nucleotide polymorphism; UKBB, UK Biobank.

a Estimates from Rome III IBS GWAS meta-analyses (UKBB and LL).

**Table 8:** Conditional GWAS Analysis on the Use of CVD and Antidepressant Medications

Risk locus		Conditional medication							
		C01D Vasodilators used in cardiac diseases	C02 Antihypertensives	C03 Diuretics	C07 Beta blocking agents	C08 Calcium channel blockers	C09 Agents acting on the renin-angiotensin system	C10AA HMG CoA reductase inhibitors	N06A Antidepressants
rs6899057 (IBS) *ADCY2*	OR	1.07 (1.04 - 1.09)							
	P	2.0E-08							
	OR-cond	1.07 (1.04–1.09)	1.07 (1.04–1.09)	1.07 (1.04–1.09)	1.07 (1.04–1.09)	1.07 (1.04–1.09)	1.07 (1.04–1.09)	1.07 (1.04–1.09)	1.06 (1.04–1.09)
	P-cond	1.2E-07	2.4E-08	9.1E-09	8.0E-09	1.2E-08	1.3E-08	1.4E-08	2.6E-07
rs6899057 (IBS-M) *ADCY2*	OR	1.09 (1.05–1.12)							
	P	2.3E-08							
	OR-cond	1.09 (1.05–1.12)	1.09 (1.05–1.12)	1.09 (1.05–1.12)	1.09 (1.06–1.12)	1.09 (1.05–1.12)	1.09 (1.05–1.12)	1.09 (1.05–1.12)	1.08 (1.05–1.11)
	P-cond	8.3E-08	2.5E-08	1.9E-08	1.4E-08	2.2E-08	2.2E-08	2.0E-08	1.1E-07
rs2048419 (IBS-M) *CLDN23* / *MFHAS1*	OR	1.08 (1.05–1.11)							
	P	4.4E-08							
	OR-cond	1.08 (1.05–1.11)	1.08 (1.05–1.11)	1.08 (1.05–1.11)	1.08 (1.05–1.11)	1.08 (1.05–1.11)	1.08 (1.05–1.11)	1.08 (1.05–1.11)	1.07 (1.04–1.10)
	P-cond	1.4E-07	1.5E-08	2.7E-08	1.8E-08	4.0E-08	3.2E-08	3.5E-08	1.0E-06
rs2035380 (IBS) *BMAL1*	OR	1.07 (1.04–1.09)							
	P	3.1E-08							
	OR-cond	1.07 (1.04–1.10)	1.07 (1.05–1.10)	1.07 (1.05–1.10)	1.07 (1.05–1.10)	1.07 (1.04–1.10)	1.07 (1.05–1.10)	1.07 (1.05–1.10)	1.07 (1.04–1.10)
	P-cond	1.8E-07	2.8E-08	1.5E-08	3.0E-09	2.5E-08	1.4E-08	1.6E-08	1.5E-07
rs9517497 (IBS) *DOCK9*	OR	1.07 (1.04–1.09)							
	P	2.3E-08							
	OR-cond	1.06 (1.04–1.09)	1.07 (1.04–1.09)	1.07 (1.04–1.09)	1.07 (1.04–1.09)	1.07 (1.04–1.09)	1.07 (1.04–1.09)	1.06 (1.04–1.09)	1.07 (1.05–1.10)
	P-cond	5.9E-07	2.7E-08	1.4E-08	3.6E-08	2.5E-08	2.6E-08	3.8E-08	9.5E-09

NOTE. Conditional analyses used GWAS summary-level data on the use of CVD and antidepressant medications, obtained from Wu et al^21^ (PMID:31015401).

CoA, coenzyme A; CVD, cardiovascular diseases; GWAS, genome-wide association study; HMG, 3-hydroxy-3-methylglutaryl; IBS, irritable bowel syndrome; IBS-M, irritable bowel syndrome mixed subtype; OR, odds ratio (with 95% confidence interval) of the associated lead SNP for the discovery IBS and IBS-M GWAS; OR-cond, odds ratio (with 95% confidence interval) of the associated lead SNP after conditioning IBS/IBS-M GWAS results on medication use.

### Rome III IBS Polygenic Risk Scores

Finally, we computed and tested Rome III IBS polygenic risk score (PRS), to assess the relevance and translational potential of our GWAS results in predicting IBS risk. For this purpose, in the absence of other large cohorts with Rome III and genotype data available, we used phenotype and genotype data from the independent set of UKBB participants who did not fill the digestive health questionnaire (DHQ nonresponders), and who were therefore not included in the Rome III IBS GWAS (Figure 1). In this dataset, IBS cases were identified based on hospital-inpatient records and self-reported diagnoses, whereas control subjects were the remainder of DHQ nonresponders (Methods). Two similarly sized nonoverlapping fractions (n = 146,771 and 146,772) of the DHQ nonresponder dataset were then used, respectively, for computation and testing of Rome III IBS PRS (see Table 9 for demographics).

**Table 9:** Demographics of UKBB Study Cohorts Included in PRS Analyses

Cohort		N	Age, mean (SD)	Sex, female (*%*)
Subset 1 (PRS computation)	IBS	5,335	57.0 (8.1)	73.9
	Control subjects	141,436	57.2 (8.2)	52.2
Subset 2 (PRS testing)	IBS	5,336	56.9 (8.1)	74.8
	Control subjects	141,436	57.2 (8.2)	52.1

NOTE. IBS cases and control subjects for PRS analyses were identified based on ICD10 K58 (code for IBS) and self-reported IBS diagnosis from touchscreen questionnaire (see Methods).

IBS, irritable bowel syndrome; ICD10, International Classification of Diseases, 10th revision; PRS, polygenic risk score; SD, standard deviation; UKBB, UK Biobank.

**Figure 9:**
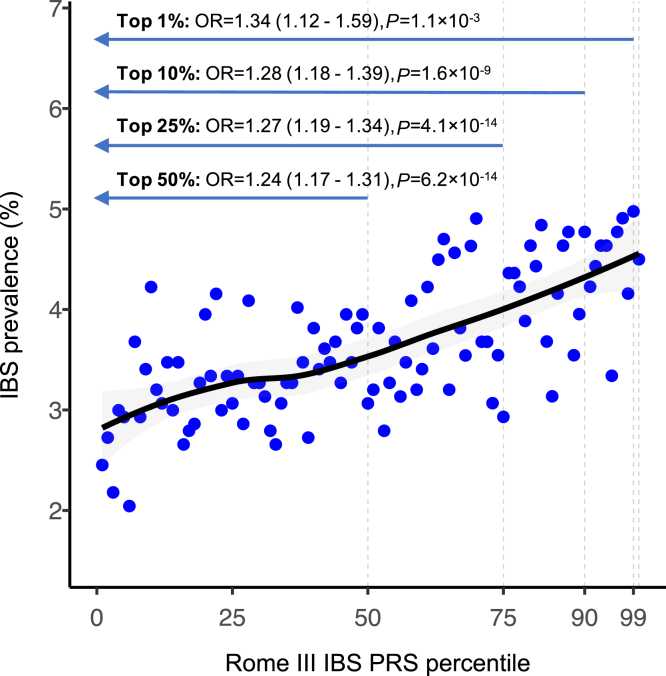
**Rome III IBS PRS testing analysis.** Prevalence of IBS across PRS percentiles (polynomial regression fitting line in *black*), and OR relative to various upper percentiles compared with the rest of the population (estimated from logistic regression adjusted for sex, 10 top principal components, and genotyping array). The testing cohort consisted of IBS cases (n = 5336) and control subjects (n = 141,436) identified based on ICD10 K58 (for IBS) and self-reported IBS diagnosis.

Using the PRSice-2^22^ pipeline (Methods), we computed a PRS model based on the inclusion of 51,509 SNP markers that were best able to differentiate IBS cases (n = 5335) from control subjects (n = 141,436) in the first dataset (*P* = 6.0×10^-13^). Using this marker set in the testing group (5336 IBS cases and 141,436 control subjects), we detected significantly higher PRS values in IBS cases compared with control subjects (*P* = 7.9×10^-22^). Notably, IBS prevalence progressively increased across the PRS distribution, reaching 4.5% in the top 1% percentile (OR, 1.34; *P* = 1.1×10^-3^ vs the rest of the population) (Figure 9).

## Discussion

We report a large-scale, population-based survey of clinical and genetic data in relation to IBS and its subtypes according to the gold standard Rome III criteria. Based on GWAS meta-analyses of 2 independent cohorts comprising 101,884 individuals of European ancestry, we show that Rome III IBS and its subtypes are heritable traits, with SNP-based heritability ranging between 10% and 13%. This is higher than 5.8% SNP heritability reported in previous larger IBS GWAS meta-analyses of multiple heterogeneous IBS definitions,^15^ suggesting that standardized Rome III criteria might better capture the contribution of core common genetic risk factors to IBS. The incomplete overlap of GWAS associations signals detected here and in previous studies may also indicate that different definitions of IBS (ICD10 diagnoses from electronic medical records, self-reported doctor diagnoses, Rome criteria, or all combined) may capture slightly different genetic underpinnings.^9^ At the same time, the genetic architecture of Rome III IBS is largely shared across subtypes, including those at the opposite end of the stool consistency spectrum (as shown in genetic correlations of IBS-D and IBS-C). Rome III IBS also shows considerable genetic overlap with other GI, psychiatric, and extraintestinal pain-related conditions that, as shown in previous studies,^23^^,^^24^ are often comorbid as in our UKBB ICD10 analyses. Novel from this study is the observation of strong genetic correlation between IBS and heart-related conditions and traits, including family history of heart disease and hypertension, ischemic (coronary) heart disease, and angina pectoris, ranging between 20% and 45%.

This novel link between IBS and cardiovascular traits was further consolidated using 2 independent, different analytical approaches: GWAS devoid of individuals with heart diseases, and GWAS conditioned on CVD medications (as proxy for CVD) both provided evidence that the genetic risk effects observed for Rome III IBS are not dependent on heart-related comorbid conditions, nor on their genetic underpinnings. Showing correlation with all Rome III subtypes, the genetic (sibling history of) hypertension was most pronouncedly similar to that of IBS-C. Of note, similar observations, albeit lacking a genetic basis, have been recently made in large epidemiologic surveys showing that patients with IBS-C are exposed to a 2-fold increased risk of poor cardiovascular outcomes compared with healthy individuals.^25^, ^26^, ^27^ This was evident across a series of heart-related conditions including hypertension, venous thromboembolism, angina, peripheral artery disease, myocardial infarction, stroke, and arrhythmias. In relation to the latter, it is worth noting that genetic commonalities with IBS have also been described for the *SCN5A* gene (coding for the NaV1.5 sodium channel with a pacemaker role in myocytes and gut interstitial cells of Cajal), whose rare defective variants play a role both in Brugada syndrome and IBS-C.^11^^,^^28^ In addition, other cross-mechanisms may link IBS to heart-related traits, including alterations of the autonomic/sympathetic control of BP and cardiovascular activity, as observed in patients with IBS more severe symptoms.^29^ Therefore, similar to what has been previously shown for anxiety and mood disorders, pleiotropic mechanisms seem to be important also for the predisposition to IBS and CVDs, something that is highlighted here for the first time.

Evidence linking Rome III IBS to cardiovascular traits also came from the analysis of individual GWAS signals. The 2 novel IBS risk loci identified here, led by rs2035380 on chromosome 11 (for IBS) and rs2048419 on chromosome 8 (for IBS-M), had been previously linked to hypertension, diastolic/systolic BP, and coronary artery disease (as per results publicly available in PhenoScanner and GWAS Atlas).^30^, ^31^, ^32^, ^33^ Although multiple genes were mapped to the rs2048419 locus, only 1, *BMAL1*, is associated with rs2035380. *BMAL1* (or *ARNTL*) codes for a core transcriptional factor that controls circadian rhythm and chronophysiologic body mechanisms, including digestive and cardiac functions.^34^^,^^35^ Of pharmacologic interest, *BMAL1* regulators are targeted by antibiotic drugs used to treat IBS-D and traveler’s diarrhea (eg, rifaximin, norfloxacin, and ofloxacin).^6^^,^^36^ *BMAL1*-deficient mice show altered diurnal oscillations in gut microbiota composition associated with changes in the colonic concentration of short-chain fatty acids and bile acids, microbial metabolites known to be relevant to both GI motility and BP regulation.^37^, ^38^, ^39^ In line with this, functional studies demonstrated GI clock disruption is associated with abnormal colonic motility.^40^ Altogether, these findings suggest that similar pathogenetic mechanisms may contribute to Rome III IBS and heart disease via genetic disruption of the circadian system, warranting further investigations.

Interesting observations also came from the functional annotation of the other 3 GWAS risk loci. Association signals at rs6899057 on chromosome 5 and rs9517497 on chromosome 13 had been previously reported,^15^ and correspond to single gene annotations (respectively *ADCY2* and *DOCK9*). In the context of findings from this study, it is noteworthy that *ADCY2* (adenylyl cyclase isoform II) is the target of colforsin (NKH477), a compound that has smooth muscle relaxant and vasodilation properties with indication for acute heart failure.^41^^,^^42^ Moreover, *ADCY2* mutations have been associated with congenital heart disease and abnormal calcium signaling.^43^ Of relevance to IBS, this gene acts downstream of G-protein coupled receptors, such as the muscarinic acetylcholine^44^ and serotonin (5-hydroxytryptamine)^45^ receptors, which play key roles in the autonomic control of GI motility^46^^,^^47^ and cardiovascular function,^48^^,^^49^ which are pathways targeted by current drugs used to treat IBS.^6^ Hence, AC2 may hold potential for IBS therapeutic exploitation.

At the novel rs2048419 IBS-M locus on chromosome 8 *CLDN23* and *MFHAS1* were prioritized among 5 candidate genes mapped to the region. *CLDN23* encodes the nonclassical tight-junction claudin-23 protein that has been recently demonstrated, both in vitro and in vivo, to modulate the intestinal epithelial barrier.^50^ This occurs via interaction of claudin-23 with other claudin isoforms (particularly claudin-3 and -4) at the luminal surface of intestinal epithelial cells, leading to regulation of paracellular permeability to ions and macromolecules and strengthening of barrier function.^50^ Changes in the expression of other claudins have been documented in patients with IBS (especially IBS-D and IBS-M), and linked to severity of GI symptoms.^51^^,^^52^ Hence, it is possible that similar (still uncharacterized) genotype-drive mechanisms are responsible for the putative involvement of claudin-23 in IBS. *MFHAS1*, however, plays a role in the modulation of inflammatory responses via macrophage polarization and toll-like receptor signaling (particularly toll-like receptor 2 and toll-like receptor 4).^53^, ^54^, ^55^ The latter drives inflammation on recognition of microbial components (eg, lipopolysaccharide), and multiple studies report increased toll-like receptor expression in the intestinal mucosa of patients with IBS.^56^^,^^57^ The interplay between gut barrier disruption and immune overactivation is hypothesized to be linked to IBS progression via sensory afferent overstimulation, resulting in pain dysfunctions.^51^ Interestingly, *CLDN23* and *MFHAS1* are expressed in intestinal enteroendocrine cells, which are key sensors of the intestinal environment and play important roles in modulating gut-brain sensory and immune responses.^58^ Additional genetic and functional studies are needed to unequivocally establish weather *CLDN23* or *MFHAS1* or both contribute to IBS pathophysiology.

Rome III IBS GWAS data were also tested for their potential to contribute to IBS risk prediction, via PRS computation and testing. Despite the lack of accurate Rome III classifications in the independent UKBB dataset used for this purpose, our PRS model still allowed the identification of individuals at higher risk of IBS (self-reported or from hospital admissions), with those in the top 1% of PRS distribution being exposed to more than 1.3 times higher risk of disease. Similar results were recently obtained using stool frequency PRS to capture IBS in UKBB,^14^ which collectively indicates that, once refined and integrated with other predictive markers, IBS PRS may find translational scope for clinical utility: they may contribute to algorithms designed for differential diagnosing GI diseases (eg, IBS vs inflammatory bowel disease) and/or the decision-making process associated with costly and/or invasive procedures (endoscopy), with positive repercussions on the health care system and the patient.

Finally, although subtype-specific analyses did not reveal major differences, we observed IBS-C was the trait that most often departed from other subtypes in its genetic correlations with other conditions, suggesting specific genetic risk factors and mechanisms may be associated with constipation. Nevertheless, no GWAS significant signals were detected for Rome III IBS-C and IBS-D subtypes: it cannot be excluded that this is caused, for instance, by the current sample size that is insufficient (ie, smaller than IBS-M) to capture weak individual risk effects. Indeed, the measured heritability of these traits seems to be slightly lower than that of IBS-M. This issue can be better addressed in follow-up, possibly larger GWAS meta-analyses in independent cohorts, or via alternative gene-hunting strategies based on the analysis of endophenotypes, such as stool frequency and consistency.^14^

Important study limitations need to be acknowledged, including: (1) having studied IBS defined according to the older version of the Rome III Criteria, which warrants evaluation of current findings in patients defined according to stricter Rome IV (or possibly upcoming Rome V) criteria when suitable large cohorts with these data become available; (2) GWAS analyses not performed for ancestries other than European; (3) minimal understanding of the pleiotropic mechanisms linking IBS to cardiovascular traits; (4) sample size still likely inadequate to fully capture genetic liability for IBS subtypes; and (5) PRS testing performed on suboptimal IBS classifications because of the lack of other suitable Rome III IBS cohorts with genetic data available.

In summary, we show that IBS types defined according to Rome III criteria are heritable traits whose genetic architecture is shared with other polygenic traits, including CVDs highlighted here for the first time. We also identify novel candidate genes and plausible mechanisms that represent potential actionable targets warranting further investigation in follow-up studies.

## Materials and Methods

The study design is summarized in Figure 1. Detailed methodologic description is provided below.

### Study Cohorts and Definition of Rome III IBS Cases and Control Subjects

#### UK Biobank

The UKBB is a population-based longitudinal cohort of >500,000 UK individuals (aged 40–69 years) with available genotype, demographic, and health-related data.^59^ On exclusion of individuals of non-White ethnic background (data field 21000), sex mismatches (data fields 31 and 22001), and low-quality genotypes (data field 22010), we focused on data from the DHQ (which includes a Rome III module for IBS), available for a subset of UKBB participants who responded to the invitation to participate in this follow-up. After exclusion of all individuals with other potentially confounding GI diagnoses that could result in IBS-like symptoms, including celiac disease, inflammatory bowel disease (and others listed in Table 10), patients with IBS (N = 22,745 total; 12,335 IBS-M; 5920 IBS-D; 3855 IBS-C) were identified using Rome III DHQ questions (data fields 21025, 21027, 21028, 21031–21034). Asymptomatic control subjects (n = 66,631) were defined as the remainder of the population without abdominal pain/discomfort in the previous 3 months (data field 21025) and without IBS, based on IBS diagnoses from hospital inpatients records (ICD10 K58 code; data fields 41202 and 41204) and self-reported IBS from questionnaire data (data fields 21024 and 20002). The study was approved by the Monash University Institute Research Ethics Committee with protocol number 20326 and performed using UKBB data accessed under application number 17435.

**Table 10:** Exclusion Criteria Applied for UKBB IBS Cases and Control Subjects

Data-field		Trait
ICD10	41202 and 41204	K50 (Crohn's disease)
		K51 (Ulcerative colitis)
		K52 (Other and unspecified noninfective gastroenteritis and colitis)
		K86 (Other diseases of pancreas)
		K90 (Intestinal malabsorption)
		K550 (Acute vascular disorders of intestine)
		K551 (Chronic vascular disorders of intestine)
		K627 (Radiation proctitis)
		C15 (Malignant neoplasm of esophagus)
		C16 (Malignant neoplasm of stomach)
		C17 (Malignant neoplasm of small intestine)
		C18 (Malignant neoplasm of colon)
		C19 (Malignant neoplasm of rectosigmoid junction)
		C20 (Malignant neoplasm of rectum)
		C21 (Malignant neoplasm of anus and anal canal)
		C22 (Malignant neoplasm of liver and intrahepatic bile ducts)
		C23 (Malignant neoplasm of gallbladder)
		C24 (Malignant neoplasm of other and unspecified parts of biliary tract)
		C25 (Malignant neoplasm of pancreas)
		C26 (Malignant neoplasm of other and ill-defined digestive organs)
		K57 (Diverticular disease of intestine)
		K58 (Irritable bowel syndrome)^a^
Self-report	20002	1462 (Crohn’s disease)
		1463 (Ulcerative colitis)
		1459 (Colitis / not Crohn’s or ulcerative colitis)
		1461 (Inflammatory bowel disease)
		1164 (Pancreatic disease)
		1165 (Pancreatitis)
		1456 (Malabsorption/coeliac disease)
		1191 (Gastrointestinal bleeding)
		1509 (Gastroenteritis/dysentery)
		1600 (Bowel / intestinal perforation)
		1601 (Bowel / intestinal infarction)
		1602 (Bowel / intestinal obstruction)
		1135 (Stomach disorder)
		1458 (Diverticular disease / diverticulitis)
		1562 (Food intolerance)
		1154 (Irritable bowel syndrome)^a^
DHQ	21068	Positive answer for "have you been diagnosed with coeliac disease/gluten sensitivity?"
	21024	Positive answer for "have you ever been diagnosed with IBS?"
	21025	Different answer than 'never' for "in the last 3 months, how often did you have discomfort or pain anywhere in your abdomen?"^a^
	21025–21034	Fulfilling Rome III criteria for irritable bowel syndrome^a^

DHQ, Digestive Health Questionnaire; IBS, irritable bowel syndrome; ICD10, International Classification of Diseases,10th Edition; UKBB, UK Biobank.

a Exclusion criterion applied to control subjects only.

#### Lifelines

LL is a multidisciplinary prospective population-based cohort study examining in a unique 3-generation design the health and health-related behaviors of 167,729 persons living in the North of the Netherlands.^60^ It uses a broad range of investigative procedures in assessing the biomedical, sociodemographic, behavioral, physical, and psychological factors that contribute to the health and disease of the general population, with a special focus on multimorbidity and complex genetics. In this study, we used health-related and genotype data for IBS GWAS analyses in an LL subset with ethical approval for genetic studies. Similar to UKBB, 1990 IBS cases (797 IBS-M, 561 IBS-D, and 475 IBS-C) and 10,518 asymptomatic control subjects were identified according to the Rome III criteria from digestive questionnaire data. The LL protocol was approved by the UMCG Medical ethical committee with protocol number 2007/152.

### Comorbidity Analysis in UKBB

For comorbidity analysis, we analyzed 250 UKBB ICD10-related data-fields (from 41202-0.0/0.65 to 41204-0.0/0.183) across 89,376 individuals, totaling 2,234,400 data-points. We tested 1116 ICD10 traits, coded for diseases (chapters I to XIV) and laboratory findings (chapter XVIII), for their differential risk across Rome III IBS cases and asymptomatic control subjects. Associations for the number of recorded ICD10 comorbidities (excluding K58 for IBS) were tested in a linear regression model, adjusting for sex and age in R v3.6.2 (https://www.r-project.org/). ORs were calculated based on logistic regression models (also adjusted for age and sex) at 2 ICD10 levels: chapters (hierarchically subdivided into domains; see https://icd.who.int/browse10/2019/en#); and 3-digit code level (including only 96 conditions with ≥1% of prevalence in the DHQ subset of UKBB). To control for type I error, the Benjamini-Hochberg false discovery rate (FDR) method was applied in the calculation of statistical significance for multiple tests correction (corrected α=0.05).

### Genotyping, Quality Control, and Genotype Imputation

Similar quality control pipelines and imputation methods were applied to both UKBB and LL cohorts, and are described as follows.

#### UK Biobank

Genome-wide genotyping of UKBB participants was carried out using custom Axiom (Affymetrix UK Biobank and UK BiLEVE) arrays. Nongenotyped variants were imputed centrally by UKBB researchers, using Haplotype Reference Consortium, UK10K, and 1000 Genomes (1KG) as reference panels. Genetic quality control procedures and PC analysis performed by UKBB researchers are thoroughly described by Bycroft et al.^59^ Quality control included checking for deviations from Hardy-Weinberg equilibrium, batch and plate effects, sex effects, and array effects across control replicates. Only SNPs with a call rate >0.99, imputation quality score INFO ≥0.9, minor allele frequency ≥0.01, nonindels, nonmultiallelic, and in autosomal chromosomes were tested in association analyses, adjusted for the top 10 PCs (data field 22009). Only individuals with White European ethnic background (categorized as “British,” “Irish,” or “Any other White background”; data field 21000) and without poor heterozygosity and missing rates (data field 22010) were included, leaving 22,745 Rome III IBS cases and 66,631 asymptomatic control subjects for GWAS analyses.

#### Lifelines

Genome-wide genotyping of LL participants was carried out within the UMCG Genotyping Lifelines Initiative. A total of 38,020 participants were genotyped using Infinium Global Screening Array Multi-ethnic Disease Version 1.0 (Illumina).^60^ Quality controls were based on checking Hardy-Weinberg equilibrium and call rate >0.95 using PLINK 1.9, sex and expected relationships from pedigree, and PC analysis to check for population outliers (as described in http://wiki-lifelines.web.rug.nl/doku.php?id=ugli). Before imputation, genotypes were prephased using SHAPEIT2^61^ and aligned to the reference panels using Genotype Harmonizer^62^ to resolve strand issues. Imputation was performed with Beagle 3.1.0^63^ and Minimac,^64^ based on whole-genome sequencing data from the Haplotype Reference Consortium reference panel. Only SNPs with INFO ≥0.9, minor allele frequency ≥0.01, nonindels, nonmultiallelic, and in autosomal chromosomes were tested in 1990 samples identified as IBS cases and 10,518 asymptomatic control subjects.

### Rome III IBS GWAS and Meta-Analyses

Rome III IBS (and subtypes) GWAS analyses were performed with a logistic mixed model using SAIGE v0.42.1^65^ adjusted for age, sex, 10 top PCs, and array (only for UKBB analyses). Individual IBS subtype GWAS summary statistics were inspected with the R package EasyQC v9.0,^66^ to check for data integrity, remove invalid or unmapped markers, and harmonize SNP identifiers (rs IDs) and allele strand coding across datasets. Markers with allele mismatches or with allele frequency deviating >0.2 compared with the Haplotype Reference Consortium reference panel were excluded. IBS-subtype specific meta-analyses were performed using a fixed-effect inverse-variance-based analysis with METAL v2011-03-25,^67^ including 6,311,313 markers in common between UKBB and LL individual GWAS. A Cochran Q test was performed to assess heterogeneity of effect-size estimates between individual GWAS, and markers with a P-Het <0.05 were excluded from subsequent subtype-specific analyses.

### Bayesian Fine-Mapping Analysis

A Bayesian fine-mapping analysis was carried out using FINEMAP v1.4^68^, to determine a minimum set of variants (credible set) containing likely causal variants for each associated risk locus. Posterior inclusion probability was estimated for each credible variant. As input, all genetic variants located within each risk locus were extracted, and the local linkage disequilibrium (LD) structure was calculated using genotypes from European UKBB individuals as reference.

### Annotation of GWAS Risk Loci and Gene Mapping

For annotation of GWAS risk loci and gene mapping, we used the online tool FUMA v1.3.5^69^ (https://fuma.ctglab.nl/tutorial). All loci at genome-wide significance (*P* ≤ 5×10^-8^) were annotated by FUMA, with lead SNPs being defined as those with the lowest *P* values in each genomic risk locus. Manhattan plots were produced using the R package qqman^70^ and Locuszoom^71^ was used for regional visualization of risk loci. Independent significant SNPs were identified based on LD with the lead SNPs (*R*^2^>0.6, using 1KG-EUR as reference panel) and *P* value (*P* ≤ 5×10^-8^). The maximum distance between LD blocks to merge into a locus was set to 250 kb. Gene mapping at each risk locus was based on position (distance <10 kb) and cis eQTL (p_FDR_ < .05) analyses, excluding the major histocompatibility complex region, in view of its LD complexity. The analyzed eQTL database included 54 tissues from GTEx v8^72^ (https://gtexportal.org/).

### SNP Heritability and Genetic Correlation Analyses Via LDSC

SNP-based heritability (h^2^_SNP_) and genetic correlations (r_g_) with other 1400 traits were estimated using LDSC,^73^ as implemented in the CTG-VL^74^ platform. Traits with <1000 cases, <5000 overall participants, and duplicated were removed. *P* values were adjusted for multiple comparisons using the FDR method (corrected α=0.05).

### Phenome-Wide Association Study

For each IBS risk locus, lead SNPs and/or their LD proxies (*R*^2^ >0.8) were used to screen for previously reported GWAS signals (*P* ≤ 5×10^-8^), inspecting data from OpenTargets,^75^ PhenoScanner v2,^32^ and GWAS ATLAS.^33^ Domains associated with individual GWAS findings were harmonized according to GWAS ATLAS classifications.

### Prioritization of Candidate Genes

For the novel risk locus with multiple candidate genes (rs2048419), we attempted to identify those most likely to play a causative role based on functional evidence from fine-mapping (FINEMAP^68^), eQTL and gene expression (GTEx^72^ and Human Protein Atlas^76^), GWAS databases (OpenTargets,^75^ PhenoScanner v2,^32^ and GWAS ATLAS^33^), DrugBank (https://go.drugbank.com/), and literature mining. Genes were prioritized based on the following criteria: (1) considerable expression (set as log_2_ transcript per million ≥3) in GTEx tissues of interest (GI and/or brain), (2) credible SNP-eQTL colocalization in tissues of disorder of gut–brain interaction interest (GI and/or brain), (3) known association with relevant traits from previous GWAS studies, (4) plausible mechanistic link to IBS pathophysiology; and (5) relevant phenotype from knockout/knockdown models or human studies.

### Rome III IBS Risk Loci Sensitivity Analysis of CVD Traits

Reevaluation of Rome III IBS loci association signals in UKBB devoid of heart-related conditions was obtained by removing from the analyses individuals with diagnoses from the ICD10 chapter IX *diseases of the circulatory system*. These included: I10 *essential (primary) hypertension*, I20 *angina pectoris*, I25 *chronic ischemic heart disease*, I44 *atrioventricular and left bundle-branch block*, I48 *atrial fibrillation and flutter*, I73 *other peripheral vascular diseases*, I83 *varicose veins of lower extremities*, I84 *hemorrhoids*, and I95 *hypotension*. As previously, association testing was carried out with SAIGE v0.42.1,^65^ adjusted for age, sex, 10 top PCs, and genotyping array. In total, 15,041 individuals with chapter IX diagnoses were removed from asymptomatic control subjects, whereas 5993 were removed from Rome III IBS cases (5993 from IBS, 3380 from IBS-M, 1475 from IBS-D, and 998 from IBS-C).

### Multitrait Conditional Analysis

Multitrait-based Conditional and Joint analysis (mtCOJO 1.93.2 beta^20^) was used to generate Rome III IBS GWAS summary statistics (outcome) conditioned on GWAS data for the use of CVD and antidepressant medications (exposures). mtCOJO estimates the effect of the exposure on the outcome either by generalized summary-data-based mendelian randomization or from genetic correlation (rg) analysis, when there are not enough LD independent (*r*^2^ <0.05) genome-wide significant SNPs for the exposure or outcome traits (at least 10 required by default). mtCOJO analyses were carried out on IBS and IBS-M GWAS meta-analyses data versus GWAS of CVD medication traits derived from Wu et al,^21^ including: C01D *vasodilators used in cardiac diseases*, C02 *antihypertensives*, C03 *diuretics*, C07 *beta blocking agents*, C08 *calcium channel blockers*, C09 *agents acting on the renin-angiotensin system*, C10AA *3-hydroxy-3-methylglutaryl coenzyme A (HMG CoA) reductase inhibitors*, and N06A *antidepressants*, according to the drug coding classification of the World Health Organization Anatomical Therapeutic Chemical Classification System.

### Polygenic Risk Score Analysis

We adopted a 2-step approach to PRS computation and testing in independent subsets of UKBB, only studying participants who did not fill the DHQ questionnaire (DHQ nonresponders) and were, thus, not included in the original Rome III GWAS meta-analyses. In the absence of Rome III data for these individuals (Rome III modules are part of the DHQ questionnaire), IBS cases were identified based on hospital-inpatient records (ICD10 diagnosis K58; data fields 41202 and 41204) and self-reported doctors’ IBS diagnosis (1154 code; data field 20002), whereas control subjects were the remainder of QCed DHQ nonresponders. Cases and control subjects were randomly assigned to 2 independent and similarly sized groups for PRS computation (5335 cases and 141,436 control subjects) and testing (5336 cases and 141,436 control subjects).

For PRS computation, according to the standard LD pruning and *P* value thresholding of PRSice-2 v2.2.11.50^22^ pipeline, varying numbers of SNPs with GWAS *P* values ranging from 5×10^-8^ to 1 were tested using the Rome III IBS GWAS summary statistics as the base file. Using default PRSice-2 pruning parameters (clump-kb=250, clump-p=1, clump-r^2^= 0.1) and 1KG-EUR LD as the reference panel, a total of 214,641 LD pruned variants entered the analyses, after exclusion of poor-quality markers (genotype missingness geno <0.1 and imputation quality INFO<0.9) and rare markers (minor allele frequency <0.01). The best Rome IBS PRS model was derived in the computation dataset, by selecting the largest and most significant Nagelkerke *R*^2^ value obtained in the *P* value thresholding strategy (*P*(*R*^2^) = 6.0×10^-13^; GWAS *P* threshold = .1056), which included 51,509 SNPs.

For the testing group, PRSs were then calculated for IBS and control subjects as the sum of effect sizes of the 51,509 markers that fitted the best Rome IBS PRS model. PRS distributions were scaled (mean, 0; standard deviation, 1) using the “scale”’ R function. A multivariate linear regression (adjusted for sex, the first 10 top genetic PCs, and UKBB genotyping array) was used to determine the significance of the difference between the mean Rome IBS PRS in IBS cases and control subjects. Individuals within a given magnitude of increased PRS in top percentiles (binned using the “ntile” dplyr R function) were compared in relation to the rest of the population for their risk to IBS in a logistic regression including previous covariates, and FDR was applied in the calculation of statistical significance for multiple tests correction.
